# Network-Based Analysis for the Quantification of Brain and Body Immune Axes with Total-Body PET Imaging

**DOI:** 10.2967/jnumed.125.271514

**Published:** 2026-05

**Authors:** Lucia Maccioni, Agne Knyzeliene, Carlos J. Alcaide-Corral, Victoria J.M. Reid, Timaeus E.F. Morgan, Martyn C. Henry, Andrew Sutherland, Mattia Veronese, Adriana A.S. Tavares

**Affiliations:** 1 Department of Information Engineering, University of Padova, Padova, Italy;; 2 Institute for Neuroscience and Cardiovascular Research, University of Edinburgh, Edinburgh, United Kingdom;; 3 Edinburgh Imaging, University of Edinburgh, Edinburgh, United Kingdom;; 4 School of Chemistry, University of Glasgow, Glasgow, United Kingdom; and; 5 Department of Neuroimaging, Institute of Psychiatry, Psychology & Neuroscience, King’s College London, London, United Kingdom

**Keywords:** animal imaging, PET, inflammation, molecular imaging

## Abstract

The brain has long been viewed as an isolated immune-privileged organ. However, growing evidence suggests a critical role of the interplay between central and peripheral inflammation in pathologic conditions. This highlights the urgent need for novel system-level analysis approaches to study the complex brain–body cross-talk during immune responses. This study aims to validate network analysis of total-body 18-kDa translocator protein (TSPO) PET imaging for studying brain and body immune axes. **Methods:** Two graph-based analysis frameworks were tested with total-body PET imaging and the third-generation TSPO radioligand [^18^F]LW223 in 2 mouse models of lipopolysaccharide-induced systemic infection (*n* = 31) and pharmacologic blocking (*n* = 19) with LW223. First, a perturbation covariance approach was adopted in the study of individualized deviation from the normative/reference interorgan immune covariance network; then, an interscan connectivity analysis was used to investigate intragroup and between-group similarities in whole-body TSPO expression patterns. **Results:** Application of the perturbation covariance approach showed increased deviations from the reference interorgan covariance network 2 h (*t*-statistic = 3.74; *P* = 8.88 × 10^−4^) and 24 h (*t*-statistic = 4.02; *P* = 4.23 × 10^−4^) after lipopolysaccharide challenge, with a 7-d recovery after infection (*t*-statistic = 0.03; *P* = 0.98), reflecting previous general evidence of a time-dependent immune response to lipopolysaccharide infection. Additionally, the perturbation approach revealed a widespread dose-specific increase in deviations after blocking agent administration, with a magnitude of deviations and several extreme deviations increasing with dose (*F*-statistic = 37.67; *P* = 2.33 × 10^−6^). Interscan connectivity analysis confirmed strong alterations from the physiologic whole-body TSPO expression pattern 24 h after the lipopolysaccharide challenge and after administration of high-dose blocking agent. **Conclusion:** To our knowledge, this study represents the first application of network analysis to total-body TSPO PET data, supporting its adoption in the study of systemic immune responses for diagnosing immune-related conditions and evaluating immune therapies.

## Visual Abstract

**Figure:**
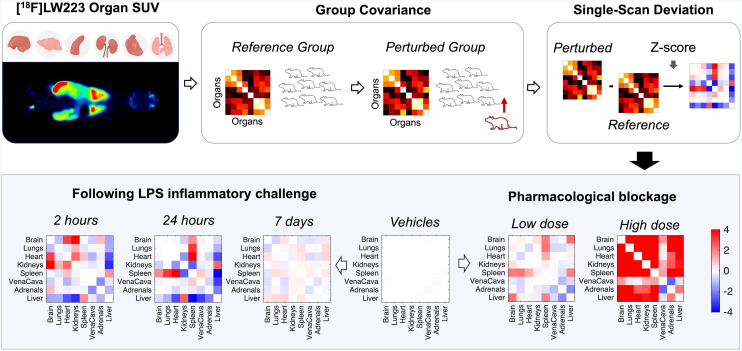

Growing evidence suggests a strong interconnection between central and peripheral inflammation (1). Several routes of communication, namely, neuronal (e.g., the sympathetic and parasympathetic branches of the autonomic system) and humoral (e.g., involving circulating inflammatory cytokines crossing the blood–brain barrier), allow for a constant cross-talk between the brain and periphery, making each one strongly affected by alterations of the homeostasis and functioning of the other (2–5). This reciprocal relationship can become particularly detrimental in pathologic conditions, including various neurodegenerative diseases, such as Alzheimer disease, Parkinson disease, and multiple sclerosis, with the bidirectional interaction exacerbating both central and peripheral inflammation (1,5–8). The loss of blood–brain barrier integrity, commonly observed in neurodegenerative disorders, can indeed facilitate the cross-passage of inflammatory cytokines and the infiltration of peripheral immune cells into the central nervous system, which may have a neurotoxic effect on the neuronal environment and promote neuroinflammation (1,3,9–11). In the opposite direction, acute brain injury promotes a systemic acute response and hepatic expression of chemokines, which induce the mobilization and recruitment of leukocytes to the brain and liver (12).

Despite increasing recognition of the role of these phenomena in the onset and progression of pathologic conditions, the relationship between central and systemic inflammation remains poorly understood, underscoring the need for system-level analytic approaches. The 18-kDa translocator protein (TSPO) in combination with PET is commonly used for neuroinflammation imaging because of TSPO upregulation in activated microglia and astrocytes (13,14). TSPO is also expressed in peripheral tissues, including the heart, lungs, liver, bone marrow, and spleen (15,16). However, whereas TSPO PET imaging has been extensively adopted to study inflammation at the single-organ level, particularly in the brain, systemic patterns of TSPO expression across multiple tissues remain underexplored (16,17). Studies have recently pointed to a potential heart–brain immune axis (18–20), further underscoring the need for new whole-body analytic perspectives. In this sense, the advent of total-body PET scanners (21), enabling the simultaneous investigation of whole-body TSPO expression, may open new opportunities to investigate systemic immune responses.

Network-based analysis of total-body PET data (22) provides a powerful multivariate analytic framework to capture this system-level interplay, allowing the examination of complex interregional relationships and the identification of potential interaction networks underlying immune responses. However, connectivity analyses of TSPO PET are still limited, and, to our knowledge, no studies have yet applied them to total-body TSPO PET data. Here, we aimed to assess the potential of network-based analysis for quantifying brain–body immune axes using total-body PET. Specifically, 2 complementary network analysis approaches were applied to [^18^F]LW223 total-body PET scans: a perturbation covariance analysis, to characterize individual deviations from a reference interorgan connectivity network (23,24), and an interscan correlation analysis, to study interscan similarity in the spatial pattern of TSPO expression. Both connectome methodologies were tested on mouse models of systemic lipopolysaccharide infection (positive network) and pharmacologic TSPO blockade (negative network).

## MATERIALS AND METHODS

### Study Animals and Imaging Experiments

Two mouse cohorts were used in this study. All animal experiments were conducted in accordance with the Home Office Animals (Scientific Procedures) Act 1986 and authorized by the local University of Edinburgh Animal Welfare and Ethical Review Committee. The ARRIVE guidelines were followed when conducting and reporting animal experiments. All animals underwent a 120-min total-body PET scan immediately after [^18^F]LW223 administration, followed by a CT scan, in the preclinical nanoPET/CT scanner (Mediso). Further details on image acquisition are reported in Supplemental Methods 1 (supplemental materials are available at http://jnm.snmjournals.org).

#### Cohort 1: Lipopolysaccharide Challenge

The first dataset included PET/CT scans on 33 mice (sex, male; age, 28 ± 2 wk, mean ± SD) after intraperitoneal injection of either 0.5 mg/kg of lipopolysaccharide from *Escherichia coli* 0111: B4 (Sigma) (*n* = 21; LPS) or vehicle solution (saline 1 mL/kg) (*n* = 12; Vehicles L). Lipopolysaccharide is a major component of the outer membrane of gram-negative bacteria, and when recognized by the immune system, it can induce a status of central or peripheral inflammation via the activation of Toll-like receptor 4 and the production of proinflammatory cytokines (25–28). Mice were scanned with the [^18^F]LW223 radiotracer (tail vein injected bolus, 10.05 ± 3.42 MBq, mean ± SD) 2 h (*n* = 3, Vehicles 2 h; *n* = 7; LPS 2 h), 24 h (*n* = 5, Vehicles 24 h; *n* = 6; LPS 24 h), and 7 d (*n* = 4, Vehicles 7 d; *n* = 8 LPS 7 d) after lipopolysaccharide or saline administration. The LPS model is a well-established model of systemic inflammation (29). Moreover, clinical assessments performed throughout the experiment confirmed the presence of significant infection-induced lethargic symptoms and substantiated the successful establishment of the model.

#### Cohort 2: Pharmacologic Blockage

The second dataset included PET/CT scans on 19 healthy C57B1/6 adult mice (sex, male; age, 15 ± 5 wk; weight, 29.17 ± 3.40, mean ± SD) after administration of either 100% dimethylsulfoxide (*n* = 3, Vehicles B) or varying amounts of nonradioactive LW223 in 100% dimethylsulfoxide as follows: 0.0009 ± 0.0004 mg/kg (*n* = 3, baseline), 0.003 ± 0.0008 mg/kg (*n* = 6, dose 1), 0.016 mg/kg (*n* = 1, dose 2), 0.22 ± 0.04 mg/kg (*n* = 3, dose 3), or 0.55 ± 0.09 mg/kg (*n* = 3, dose 4). The blocking agent was injected intravenously together with the [^18^F]LW223 bolus (9.43 ± 4.35 MBq, mean ± SD) via femoral (*n* = 12) or tail (*n* = 7) vein injection. Dimethylsulfoxide volume was maintained at the lowest feasible level and kept consistent across all animals (15.17 ± 5.73 µL). The pharmacologic blockade model, by suppressing the specific component of the PET signal, provides a valuable negative framework for testing total-body network analysis approaches.

### Image Processing and Analysis

Reconstructed PET/CT images were preprocessed and analyzed using PMOD software (PMOD Technologies), version 3.7 for cohort 1 and version 4.2 for cohort 2 (Supplemental Fig. 1). Volumes of interest (VOIs) for body organs were manually drawn from PET (kidneys, adrenal glands, spleen, liver, and vena cava) or CT images (brain, heart, and lungs). Mean VOI time–activity curves were extracted and quality checked, and 2 scans (LPS 2 h and LPS 7 d groups) were excluded because of failure in tracer injection. A whole-body VOI was drawn for the computation of the total injected dose as the sum of the whole-body radiotracer activity. VOI time–activity curves were normalized for the injected dose and animal weight to SUV, and the average SUV from 90 to 120 min (SUV_90–120_) was computed for each VOI.

### Perturbation Covariance Analysis

**FIGURE 1.**
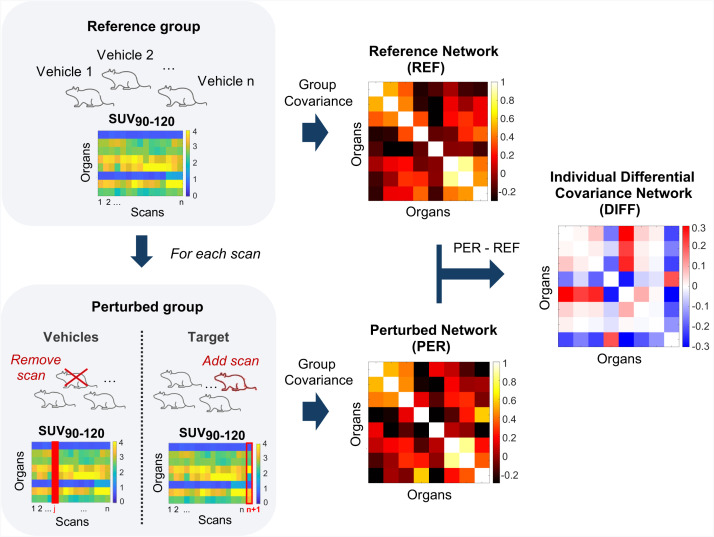
Schematic representation of perturbation covariance approach. Reference covariance matrix (REF) is computed as between-organ Pearson correlation of SUV_90–120_ across *n* Vehicles scans of reference group. Perturbed covariance matrix (PER) is then derived for each target scan by adding scan to reference group and recomputing interorgan covariance network in perturbed group. Leave-1-out approach is adopted for Vehicles mice, where perturbed group is obtained by excluding specific scan under evaluation from reference group. For each scan, differential covariance network (DIFF) is finally derived as difference between PER and REF matrices and normalized to *z* scores.

The application of the recently developed perturbation covariance approach (23,24) to the analysis of organ SUV_90–120_ (Fig. 1; Supplemental Method 2) enabled the derivation of single-subject *z*-score matrices describing individual deviations from the normative pattern of interorgan connections in a reference population. All analyses were performed using MATLAB (Matlab2023a; MathWorks). Specifically, the perturbation covariance analysis was applied to the longitudinal study of interorgan connectivity changes in TSPO expression after systemic lipopolysaccharide infection by comparison of individual deviation matrices between Vehicles L, LPS 2 h, LPS 24 h, and LPS 7 d groups (cohort 1). Additionally, the approach was applied to a target blocking study to test the specificity to TSPO by comparison of Vehicles B, baseline, doses 1–2 (including dose 1 and dose 2 scans), dose 3, and dose 4 groups (cohort 2). The same statistical framework was finally adopted to investigate possible differences between the Vehicles subgroups: Vehicles 2 h, Vehicles 24 h, and Vehicles 7 d from cohort 1 and Vehicles B from cohort 2. In all cases, an analysis of extreme deviations was performed by selecting *z* scores higher in absolute value than a critical value *Z*_threshold_ of 3.12 corresponding to a significance level α of 0.05 in a normal distribution after Bonferroni adjustment for multiple comparisons.

### Interscan Connectivity Analysis

The interscan connectivity matrix of organ SUV_90–120_ was computed in MATLAB, and, for each scan, the most similar scan in terms of the highest Pearson correlation was identified. Additionally, Graphia software was adopted for the analysis of the interscan connectivity graph, where each node represented an individual scan and edges represented their pairwise correlation. Specifically, a Markov clustering was performed to identify clusters of highly interconnected nodes in the graph, and an enrichment analysis was conducted to investigate possible enrichment of Markov clusters for scan groups. A sensitivity analysis was also performed to assess the effect of selecting different clustering granularities. Supplemental Method 3 provides additional details.

## RESULTS

### Perturbation Covariance Analysis Identified Brain, Heart, and Kidneys as First-Response Tissues, and Liver and Spleen as Second-Line Responders to Acute Systemic Infection

**FIGURE 2.**
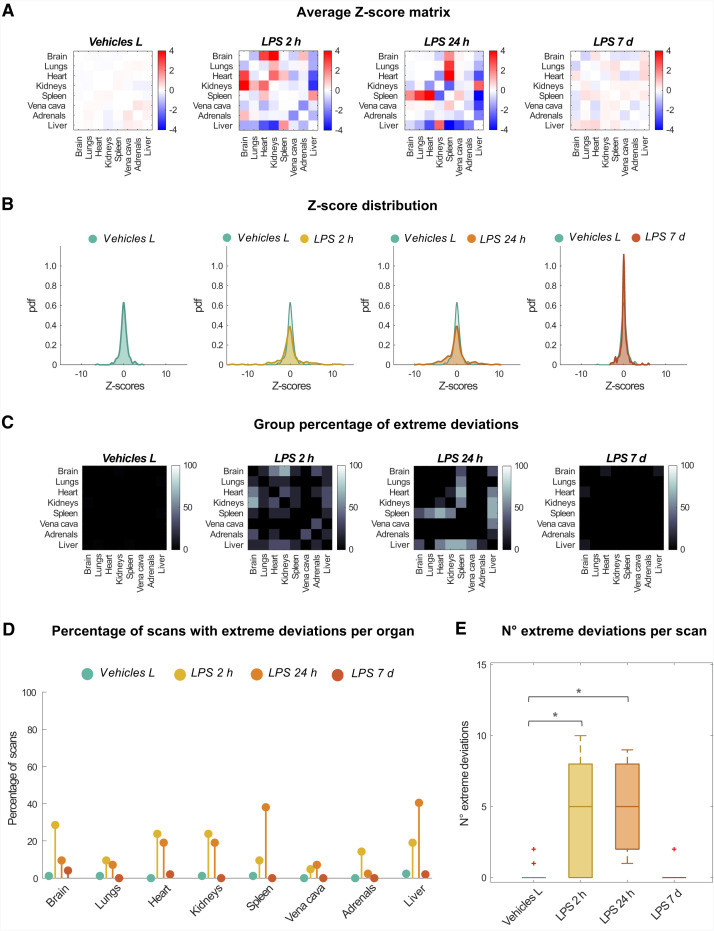
Murine whole-body perturbation analysis after systemic infection challenge with lipopolysaccharide. Figure shows, for each group, across-scans average of individual *z*-score matrices (A), probability density function (pdf) fitted over distribution of *z* scores across organs and scans (B), and extreme deviation matrices representing percentage of scans showing extreme deviations for each edge (C); percentage of scans with extreme deviations for each organ (average across organ links) (D) and number of links with extreme deviations in each scan (E) are also compared between scan groups. *P < 10^−3^; + indicates outliers.

Data showed an alteration of the normative pattern of multiorgan TSPO expression between 2 h and 24 h after lipopolysaccharide intraperitoneal administration and a recovery in the interorgan connectivity network 7 d after the challenge. Across-scans average *z*-score matrices for each group (Fig. 2A) showed negligible positive and negative values of the *z* scores for both Vehicles L (0.08 ± 0.09; mean ± SD of average *z*-score matrix absolute values) and LPS 7 d (0.27 ± 0.20) groups. Conversely, a higher magnitude of average *z* scores, for both positive and negative values, was reported for the LPS 2 h group (1.00 ± 1.10), particularly for kidneys and heart, and the LPS 24 h group (1.07 ± 1.28), particularly for spleen and liver. The same pattern of deviations across longitudinal time points was highlighted by the distributions of the *z* scores for each group (Fig. 2B), with a more platykurtic and flatter shape for the LPS 2 h and LPS 24 h distributions compared with the Vehicles group, and a sharper shape manifested by the LPS 7 d distribution. When the percentage of mice per group reporting extreme deviations for each between-organs link was examined (Figs. 2C and 2D), higher percentages were reported for the LPS 2 h and LPS 24 h, with the first group showing the highest increases for brain, heart, and kidney links (first response tissues) and the second for the liver, heart, and spleen (second line of response tissues). Overall, the strongest effects, in terms of the percentage of mice with extreme deviations, were reported for the LPS 24 h group for the liver and spleen links, suggesting these organs are critical in acute systemic responses. The percentage of extreme deviations was negligible for both Vehicles and LPS 7 d groups for almost all pairwise organ connections. An ANOVA test of group differences in the number of extreme deviations per scan (Fig. 2E) showed a statistically significant difference among the 4 groups (*P* = 2.37 × 10^−4^; *F* = 9.18). Post hoc tests revealed significant differences with respect to the Vehicles L group for the LPS 2 h (*P* = 8.88 × 10^−4^) and LPS 24 h (*P* = 4.23 × 10^−4^) but not for the LPS 7 d group (*P* > 0.05).

### Brain–Body Perturbation Analysis Can Quantify a Wide Range of Drug-Tissue and Drug-Blood Engagement in a Dose-Dependent Manner

**FIGURE 3.**
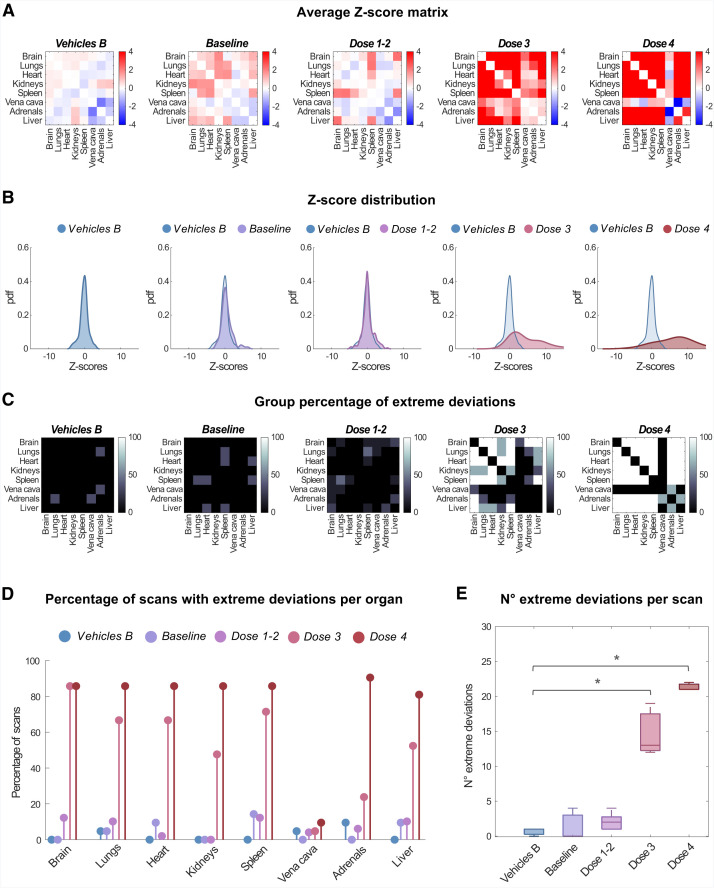
Murine whole-body perturbation analysis after intravenous administration of pharmacologic doses of nonradioactive LW223 as blocking agent. Figure shows, for each group, across-scans average of individual *z*-score matrices (A), probability density function (pdf) fitted over distribution of *z* scores across organs and scans (B), and extreme deviation matrices representing percentage of scans showing extreme deviations for each edge (C); percentage of scans with extreme deviations for each organ (average across organ links) (D) and number of links with extreme deviations in each scan (E) are also compared between scans groups. *P < 10^−6^.

Network analysis results reflected the pharmacokinetic/pharmacodynamic relationship of the blocking agent, with increasing deviations reported at increasing blocking agent doses. Group across-scans average *z*-score matrices (Fig. 3A) showed negligible *z* scores for the Vehicles B group, with both positive and negative values close to 0 (0.36 ± 0.43; mean ± SD of average *z*-score matrix absolute values), and a progressive widespread increase in magnitude of average *z* scores for baseline (0.64 ± 0.59), doses 1–2 (0.58 ± 0.71), dose 3 (4.36 ± 4.76), and dose 4 (6.42 ± 5.43) groups. The same trend was reflected in group *z*-score distributions (Fig. 3B), progressively deviating from the gaussian shape characteristic of Vehicles distribution, with a higher occurrence for high positive values. The percentage of mice in each group reporting an extreme deviation for each between-organs edge (Figs. 3C and 3D) showed a widespread dose-dependent increase, except for vena cava links. Finally, the ANOVA test reported a statistically significant group difference (*P* = 1.16 × 10^−9^; *F* = 85.00) in the number of extreme deviations per scan (Fig. 3E). Post hoc tests revealed a significant difference from the Vehicles B group for dose 3 (*P* = 2.61 × 10^−7^) and dose 4 (*P* = 1.89 × 10^−9^) but not baseline and doses 1–2 groups (*P* > 0.05).

### Multiorgan Profile Can Assign Mice to Different Experimental Groups Within an Interscan Connectivity Matrix

**FIGURE 4.**
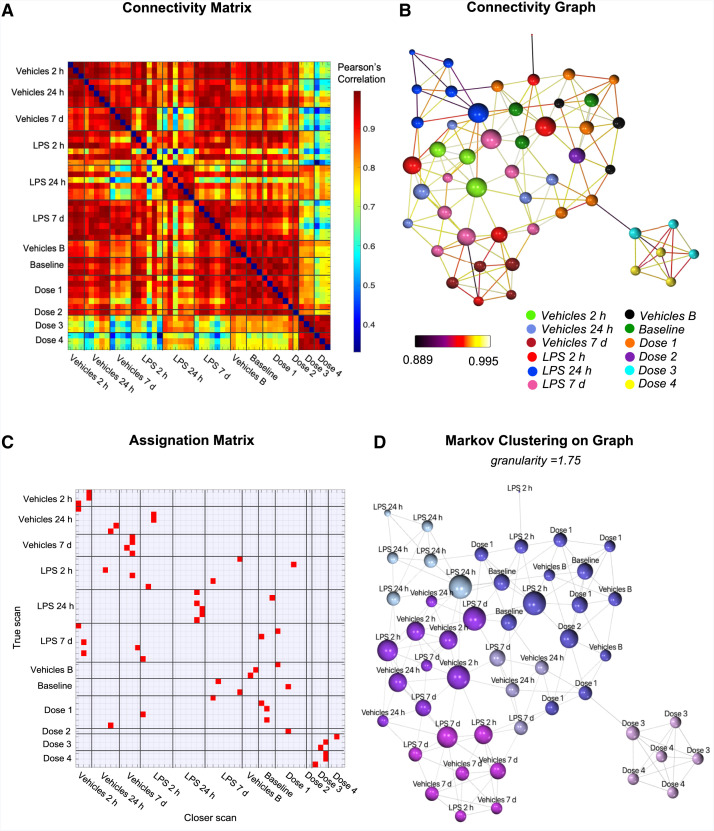
[^18^F]LW223 whole-body interscan connectivity network containing vehicle controls and test groups (lipopolysaccharide and pharmacologic blocking). (A) Connectivity matrix representing pairwise interscan Pearson correlation coefficient of SUV_90–120_ across organs. (B) Two-dimensional graph representation of interscan correlation network, with nodes representing scans (scan groups are represented with different node colors) and edges representing connection between pairs of scans (C) Matrix represents, for each scan, most similar scan in terms of higher Pearson correlation. (D) Results of Markov clustering (granularity = 1.75) performed on interscan connectivity graph with graph clusters represented by different colors.

The connectivity matrix representing pairwise interscan Pearson correlation and 2-dimensional graph representations of the interscan connectivity network are reported in Figures 4A and 4B, respectively. Dose 3 and dose 4 scans showed high intra- and intergroup similarity in terms of high Pearson correlation but showed low similarity to all Vehicles and LPS scans. Likewise, LPS 24 h scans appear highly interconnected, reflecting a common spatial pattern of TSPO expression, while showing low similarity to other groups. Conversely, all Vehicles groups, as well as LPS 7 d, baseline, dose 1, and dose 2, show high intragroup and between-groups interscan correlation of the SUV_90–120_ spatial pattern. An assignation matrix (Fig. 4C), indicating for each scan the most similar or correlated scan, confirmed the same intragroup and between-groups similarity.

The same evidence was shown by the results of Markov clustering (Fig. 4D), which identified an isolated cluster formed exclusively by dose 3 and dose 4 scans, as well as a cluster composed of LPS 24 h scans. This was also reflected by enrichment analysis (Table 1), showing a statistically significant enrichment of dose 3 and dose 4 scans in 1 of 6 identified network subclusters, as well as of LPS 24 h in a separate cluster. Dose 1 and Vehicles 7 d showed significant enrichment, as well. Similar evidence was confirmed by the sensitivity analysis of Markov clustering and enrichment analysis results to different levels of granularity selected for clustering (Supplemental Fig. 2; Supplemental Table 1).

**TABLE 1.** Enrichment Analysis on [^18^F]LW223 Interscan Connectivity Graph

Cluster	Group	*n*	Observed	Expected	Representation	Fisher *P*	Bonferroni-adjusted *P*
1	Dose 1	16	6	1.92 ± 1.29	3.13	0.0005	0.006
3	Vehicles 7 d	8	4	0.64 ± 0.78	6.25	0.0003	0.0036
4	Dose 3	6	3	0.36 ± 0.59	8.33	0.001	0.012
4	Dose 4	6	3	0.36 ± 0.59	8.33	0.001	0.012
5	LPS 24 h	6	6	0.72 ± 0.79	8.33	6.29 × 10^−8^	7.55 × 10^−7^

Table lists clusters significantly enriched for 1 or more scan groups. Representation indicates over-representation factor of specific group within 1 cluster compared with expected/average frequency. Fisher *P* represents *P* value for Fisher exact statistical test for enrichment. Bonferroni-adjusted *P* is Fisher *P* value adjusted for multiple comparisons.

## DISCUSSION

In this study, the application of 2 network-based approaches to total-body TSPO PET imaging acquired with the novel radiotracer [^18^F]LW223 revealed systemic alterations in interorgan TSPO expression interactions in mice after lipopolysaccharide administration and pharmacologic blocking. [^18^F]LW223 overcomes the drawback of previously developed TSPO PET ligands with a binding affinity that is not susceptible to TSPO rs6971 genetic polymorphism, low nondisplaceable binding, and high target affinity (18,30). Our results suggest network analysis of total-body TSPO PET imaging as a powerful tool for the study of systemic alteration in inflammatory responses in the preclinical and clinical settings to adopt in the diagnosis (via perturbation analysis), prognosis (via connectivity analysis), and treatment evaluation (via perturbation or connectivity analysis) for immune-related disorders. These methods overcome traditional univariate TSPO PET imaging approaches, which focus on detecting local increases in TSPO density, by instead examining alterations in the topology of TSPO expression throughout the body through analyses of interorgan or intersubject correlations of static organ-level TSPO PET measures.

Lipopolysaccharide is a toxic agent commonly adopted to mimic infection-induced inflammation (28,31–33). Here we have shown that brain, heart, and kidneys are the first-response tissues after acute administration of lipopolysaccharide (2 h), whereas liver and spleen involvement become more prominent 24 h after lipopolysaccharide administration. Previous studies have shown brain TSPO overexpression after lipopolysaccharide challenge in rodents, primates, and humans (34–36) as well as multiorgan TSPO PET signal alterations involving the brain, liver, lungs, and spleen (16). However, whereas these studies investigated potential increases in local neuroinflammatory load reflected by elevated TSPO density, in this work, we examined possible dysfunctions in interorgan immune interplay. To our knowledge, this study provides the first quantitative mapping of the multiorgan spatial network underlying the temporal immune response to lipopolysaccharide infection. Other evidence has suggested a dynamic modulation of immune cells response and TSPO expression over time (25,27,28,31,37–39). A direct comparison with traditional lipopolysaccharide studies is hampered by substantial differences in experimental settings, biologic factors, and body area of investigation (25,29). However, meta-analysis showed microglia activation in mice from 6 h to 3 d after a single lipopolysaccharide challenge, and no microglial activation was generally reported after this period (25). In the study by Qiu et al., lipopolysaccharide-induced infection was associated with the manifestation of depressive-like behavior 24 h after injection and symptom reduction after 72 h, which coincided with enhanced brain [^18^F]DPA-714 PET uptake and microglia activation, returning to baseline levels by 72 h (39). These results are in line with findings in rats showing an increased brain uptake 24 h after lipopolysaccharide administration (33).

Specifically, we reported strong alterations of interorgan connections involving the liver and the spleen 24 h after lipopolysaccharide administration. Experimental and clinical studies have demonstrated lipopolysaccharide’s role in liver disease, mostly mediated by tumor necrosis factor-α and interleukin-1b proinflammatory cytokines (40–46). Liver hepatocytes indeed have a primary role in the clearance of toxic agents from the intestine and gut, including lipopolysaccharide (41). On the other hand, the spleen is known to play a pivotal role in immune cell recycling (47), thereby corroborating its involvement in systemic infection responses. Changes in spleen TSPO PET uptake after lipopolysaccharide injection were also reported, likely suggesting monocytic efflux and lymphocytic activation (16). In addition, we reported substantial alterations in the heart–brain–kidneys axis 2 h after the infections. The importance of the brain–heart interaction has been increasingly recognized as a critical physiologic axis altered in disease (19,20,48). Similarly, the implication of heart–kidneys and brain–kidneys cross-talk through various bidirectional pathways in acute and chronic disease states has been highly supported (49,50). Growing evidence shows that coordinated interconnection between the brain, heart, and kidneys is essential for maintaining systemic homeostasis. This continuous communication involves multiple pathways including blood pressure control and chronic inflammation but also autophagy, oxidative stress, and Ca^2+^ signaling (51). Notably, the heart and kidneys are among the most metabolically demanding organs in the body, closely followed by the brain (52). Dysfunction in any of these regulatory mechanisms can profoundly affect not only the function of each individual organ but also their complex interaction. The brain–heart–kidneys axis hypothesis recognizes the disruption of this intricate cross-talk as a major factor in neurodegeneration and aging (51,53,54).

Methodologically, the application of the perturbation covariance method enabled the derivation from static SUV_90–120_ PET measurements of single-subject matrices of deviation from the normative pattern of connections. Connectivity analysis of static PET generally adopts a population-based approach on the basis of the computation of across-scans interorgan covariance (55). Scan-level network analysis approaches, instead, typically exploit dynamic PET data to compute the interorgan time–activity curve correlation (56). The perturbation method, although not providing pure functional connectivity information (57), still allows an individual-level analysis of deviations from the normative immune network linked to an inflammatory condition, only requiring static PET data (24). This method overcomes potential limitations of group-level approaches, where disease-related effects can be hindered by the interindividual variability in disease manifestation within the study cohorts. However, this approach requires the reliable characterization of the normative connectivity network in a reference cohort as an essential prerequisite for the accurate and robust definition of individualized deviation maps. In this study, to increase sample size, the reference group was formed by pooling all Vehicles scans (control scans) from the 2 cohorts under study. As expected, the percentage of extreme deviations was negligible (below 5%) for all Vehicles subgroups and showed no statistically significant differences between the groups (Supplemental Fig. 3).

Notably, only male mice were included in this study. Animal studies in biomedical research are often conducted on a single sex, typically males, to reduce biologic variability and simplify data interpretation (29,58,59). However, recent studies have shown multiorgan sex differences in murine TSPO (60), thus increasing concerns on the generalizability of the results across sexes. This paper represents an exploratory methodologic study primarily aimed at the validation of network analysis approaches for the study of the systemic immune responses with total-body TSPO PET data. However, future work should investigate the generalizability of study outcomes to female cohorts as well as between-sex differences in the interorgan immune network. Importantly, different mice were scanned at the selected time points. This operative choice was driven by technical and practical challenges in maintaining animals at a high level of sickness under anesthesia during the 120-min image acquisition. This choice is also in agreement with the 3R principles when developing new imaging biomarkers, where a stepwise approach is recommended. Future studies using the longitudinal design are warranted based on the positive results from our seminal study. As an additional limitation, the potential impact of tracer metabolism was not investigated. The [^18^F]LW223 radiometabolism in naïve mice, that is, the same species used in this study, is known to be low, with an average of 83% parent in plasma up to 120 min after injection (30). Therefore, the impact of radiometabolism on [^18^F]LW223 murine PET studies presented here is expected to be low. Furthermore, we have previously shown that network analysis of total-body PET mouse studies is insensitive to scan length, noise, and normalization methodology used (61). Therefore, we expect it to also be less susceptible to metabolic changes compared with traditional methods of quantification. Notwithstanding, future work specifically testing the impact of metabolism on network analysis would be worthwhile, as peripheral metabolism may impact the correlative association between the brain and other organs due to radiotracer stability variability.

Importantly, the pharmacologic blockade with LW223 does not induce a systemic immunosuppression. However, the LW223 blocking dataset offers a valuable negative-control framework in which TSPO-specific binding is selectively reduced to test the network-analysis approaches used in this study and assess their specificity for PET-related signal correlation changes.

## CONCLUSION

We validated 2 network analysis approaches for the system-level study of inflammation. Network analysis of total-body TSPO PET data showed time-dependent alterations in organ clusters showing a coordinated TSPO PET signal, identifying the brain–heart–kidneys and liver–spleen clusters as first-line and second-line responders to acute systemic infection, respectively. Under proper validation, these approaches are directly translatable to inflammation studies in humans, exploiting the advantages of emerging total-body PET scanners in the clinical environment.

## DISCLOSURE

This publication has been made possible in part by CZI grant DAF2021-225273 and grant DOI https://doi.org/10.37921/690910twdfoo from the Chan Zuckerberg Initiative DAF, an advised fund of Silicon Valley Community Foundation (funder DOI 10.13039/100014989), the MUR PNRR National Center for HPC, BIG DATA AND QUANTUM COMPUTING, the Ministry of University and Research within the Complementary National Plan PNC DIGITAL LIFELONG PREVENTION-DARE, the Fondo per il Programma Nazionale di Ricerca e Progetti di Rilevante Interesse Nazionale. This work was supported by the British Heart Foundation (RE/13/3/30183, RG/16/10/32375) and the Rosetrees Trust (Seedcorn2020\100304). Agne Knyzeliene’s studentship was supported by a Principal’s Career Development Award. Adriana Tavares, Victoria Reid, and Timaeus Morgan were funded by the British Heart Foundation (RG/16/10/32375, FS/19/34/34354). Martyn Henry was funded by an EPSRC PhD studentship (EP/M508056/1). Adriana Tavares is a recipient of a Wellcome Trust Technology Development Award (221295/Z/20/Z) and a Chan Zuckerberg Initiative DAF grant (2020-225273). Lucia Maccioni is supported by Fondo per il Programma Nazionale di Ricerca e Progetti di Rilevante Interesse Nazionale (PRIN) (project no. 2022RXM3H7). Mattia Veronese is supported by EU funding within the MUR PNRR “National Center for HPC, BIG DATA AND QUANTUM COMPUTING (project no. CN00000013 CN1), the Ministry of University and Research within the Complementary National Plan PNC DIGITAL LIFELONG PREVENTION-DARE (Project no PNC0000002_DARE), and by Fondo per il Programma Nazionale di Ricerca e Progetti di Rilevante Interesse Nazionale (PRIN) (project no. 2022RXM3H7). A patent for TSPO binders has been submitted (applications GB1810312.7 and PCT/EP2019/066546). No other potential conflict of interest relevant to this article was reported.

## Notes

KEY POINTS **QUESTION:** Can total-body PET network analysis be used to characterize immune response with multiorgan involvement, to better diagnose, prognose, and treat patients? **PERTINENT FINDINGS:** Network analysis can identify immune response time courses in multiorgan systems, where first-response tissues (2 h after lipopolysaccharide administration) identified through perturbation analysis were the brain, heart, and kidneys, and the second immune response was significant for the liver, heart, and spleen (24 h after lipopolysaccharide administration). Data also showed that responses of multiorgan axes to systemic drug administration were dose- and tissue-dependent, in which the lungs, heart, spleen, and liver were most perturbed by low drug doses, and the brain, lungs, spleen, adrenal glands, and liver perturbations were by medium drug doses; finally, all organs were perturbed by high drug doses. **IMPLICATIONS FOR PATIENT CARE:** Following translation to humans, results from this study could be used to understand how different diseases spread to multiple organ systems over time, providing better whole-person diagnostic and prognostic tools, as well as support drug discovery programs by evaluating multiple-tissue responses to varying doses of the drugs, thereby identifying tissues at greater risk of perturbation and potentially adverse outcomes.
